# Burden of depressive symptoms and non-alcohol substance abuse; and their association with alcohol use and partner violence: a cross-sectional study in four sub-Saharan Africa countries

**DOI:** 10.1017/gmh.2018.22

**Published:** 2018-10-02

**Authors:** F. Bajunirwe, S. Maling, H.-O. Adami, I. O. Ajayi, J. Volmink, C. Adebamowo, C. Laurence, T. Reid, J. Nankya-Mutyoba, F. S. Chiwanga, S. Dalal, M. Njelekela, D. Guwatudde, M. D. Holmes

**Affiliations:** 1 Department of Community Health, Mbarara University of Science and Technology, P.O. Box 1410, Mbarara, Uganda; 2 Department of Psychiatry, Mbarara University of Science and Technology, P.O. Box 1410, Mbarara, Uganda; 3 Department of Epidemiology, Harvard T.H. Chan School of Public Health, Boston, MA, USA; 4 Department of Medical Epidemiology and Biostatistics, Karolinska Institutet, Stockholm, Sweden; 5 Department of Epidemiology and Medical Statistics, Faculty of Public Health, College of Medicine, University of Ibadan, Ibadan, Nigeria; 6 The South African Cochrane Centre, South African Medical Research Council, Cape Town, South Africa; 7 Greenebaum Cancer Center and Institute of Human Virology, University of Maryland School of Medicine, Baltimore, Maryland, USA; 8 Institute of Human Virology, Abuja, Nigeria; 9 Centre for Evidence-Based Health Care, Stellenbosch University, Cape Town, South Africa; 10 Department of Epidemiology & Biostatistics, Makerere School of Public Health, Kampala, Uganda; 11 Department of Internal Medicine, Muhimbili National Hospital, Dar es Salaam, Tanzania; 12 Department of Physiology, Muhimbili University of Health and Allied Sciences, Dar es Salaam, Tanzania; 13 Channing Division of Network Medicine, Department of Medicine, Brigham and Women's Hospital and Harvard Medical School, Boston, Massachusetts, USA

**Keywords:** mental health, prevalence, sub-Saharan Africa, substance abuse

## Abstract

In sub-Saharan Africa, there are limited data on burden of non-alcohol substance abuse (NAS) and depressive symptoms (DS), yet potential risk factors such as alcohol and intimate partner violence (IPV) are common and NAS abuse may be the rise. The aim of this study was to measure the burden of DS and NAS abuse, and determine whether alcohol use and IPV are associated with DS and/or NAS abuse. We conducted a cross-sectional study at five sites in four countries: Nigeria (nurses), South Africa (teachers), Tanzania (teachers) and two sites in Uganda (rural and peri-urban residents). Participants were selected by simple random sampling from a sampling frame at each of the study sites. We used a standardized tool to collect data on demographics, alcohol use and NAS use, IPV and DS and calculated prevalence ratios (PR). We enrolled 1415 respondents and of these 34.6% were male. DS occurred among 383 (32.3%) and NAS use among 52 (4.3%). In the multivariable analysis, being female (PR  =  1.49, *p*  =  0.008), NAS abuse (PR  =  2.06, *p*  =  0.02) and IPV (PR  =  2.93, *p* < 0.001) were significantly associated with DS. Older age [odds ratio (OR) = 0.31, *p* < 0.001)], female (OR = 0.48, *p*  =  0.036) were protective of NAS but current smokers (OR = 2.98, *p* < 0.001) and those reporting IPV (OR  =  2.16, *p*  =  0.024) were more likely to use NAS. Longitudinal studies should be done to establish temporal relationships with these risk factors to provide basis for interventions.

## Background

Globally, the burden of mental illness is largely unknown and data from modeling may be underestimates (Vigo *et al*. 2016) and may be similar to cardiovascular disease in terms of disability-adjusted life-years (DALY). The most recent analysis from the Global Burden of Disease ranks mental health among the five leading causes of disease burden (GBD 2016 Disease and Injury Incidence and Prevalence Collaborators 2017). In sub-Saharan Africa, data on mental illness are very limited but existing studies indicate depression and substance abuse as two of the major mental health conditions on the continent (GBD 2016 DALYs & HALE Collaborators, 2017).

In sub-Saharan Africa, the majority of data on mental illness are from HIV-infected populations and a recent meta-analysis showed a prevalence of depression among HIV-infected people to be between 9% and 32% (Bernard *et al*. 2017). Most of these studies assess only severe depression. For instance, in recent studies among HIV-infected older adults in rural Uganda and South Africa, the prevalence of major depressive disorder (MDD) was 10% and 30%, respectively (Nyirenda *et al*. 2013; Kinyanda *et al*. 2016). Among the general population seeking HIV testing in South Africa, the prevalence of MDD was 14% (Kagee *et al*. 2016). Because these studies have been conducted among HIV-infected populations, they do not represent the general population. Because depression occurs two to three times more frequently among HIV-infected compared with the general population (Ciesla & Roberts, 2001), more studies need to be done there to obtain more generalizable information.

Alcohol use is high in sub-Saharan Africa (Ferreira-Borges *et al*. 2017), while use of non-alcohol substances (NAS) such as marijuana and methamphetamines is on the rise (Mnyika *et al*. 2011) and both may be linked to depression (Watt *et al*. 2015; Berg *et al*. 2017). However, the evidence is inconclusive, hence more data are required to shed light on the possible causal association. In addition, the majority of studies on mental illness have been conducted in health facility settings. However, the stigma of mental illness in Africa (Murray *et al*. 2015; Reta *et al*. 2016) leads significant numbers of sufferers not to seek care in formal health facilities. This suggests that estimates on conditions such as depression and NAS abuse from health facility-based studies may underestimate the true disease burden in the population. Population-based studies that can provide more accurate estimates of disease burden and risk factors are needed.

Health systems in sub-Saharan Africa need data to guide the design and implementation of mental health interventions. For instance, it is important to identify risk factors and risk groups for depression and substance abuse and reach them through interventions. Sub-Saharan Africa is experiencing demographic transitions (Pezzulo *et al*. 2017), and although overall the populations are still predominantly youthful, longevity is increasing. Therefore, the aim of this study was to estimate the burden of depressive symptoms (DS) and NAS use; and to determine whether alcohol use and IPV are associated with DS and/or NAS use in four countries in sub-Saharan Africa.

## Methods

### Study design and population

We conducted a cross-sectional study at five sites in four countries in sub-Saharan Africa in a partnership between the Harvard T. H. Chan School of Public Health, USA; the Institute for Human Virology, Nigeria; Makerere University School of Public Health, Uganda; Mbarara University of Science and Technology, Uganda; Muhimbili University of Health Sciences, Tanzania; and Stellenbosch University, South Africa called the Africa/Harvard School of Public Health Partnership for Cohort Research and Training (PaCT). The study was an initial step in a pilot testing methodologies for the feasibility of a large long-term multi-country cohort study (Holmes *et al*. 2010). Details of the structure of this partnership have been published elsewhere (Dalal *et al*. 2015). Briefly, we enrolled health care professionals at two hospitals in Nigeria, teachers employed at public schools in South Africa, primary school teachers in Tanzania, residents in a peri-urban community in the central part of Uganda and residents in a rural community in the western part of Uganda.

### Sampling and eligibility criteria

At all sites, we used a sampling frame of eligible participants to randomly select the study participants and the summary is presented in Table 1 below. Participants were eligible if they were adults aged 18 years or older whilst the professionals – teachers and health workers – should be currently employed at the time of data collection. The rural and peri-urban sites in Uganda enrolled current residents of the selected villages who did not plan to migrate to another location within 6 months. A more detailed description of the sampling strategy used at each country site has been described elsewhere (Dalal *et al*. 2015).

**Table 1.** Summary of population type and sampling procedures at the study sites.

Site	Population type	Sampling mechanism
Uganda rural	Village residents enrolled at household level, western Uganda	Simple random sampling using household lists from villages as sampling frame
Uganda peri-urban	Village residents enrolled in a survey at household level, central Uganda	Simple random sampling using household lists as sampling frame
Nigeria	Health care workers professionals at two hospitals in Abuja, one urban and another peri-urban	Simple random sampling using hospital rosters as sampling frame
Tanzania	Primary school teachers in Temeke, Dar es Salaam	Simple random sampling of schools in Temeke district of Dar es Salaam and questionnaires were sent via mail to the selected schools
South Africa	Primary and secondary teachers at public schools, Cape Town	Simple random selection of public schools in Cape Town, South Africa. Questionnaires were sent via mail to the head teachers

In Dar es Salaam, Tanzania and Cape Town, South Africa, questionnaire and informed consent packages were delivered to principals after confirming the number of teachers employed at each school. Principals were requested to distribute these packages to all the teachers. The teachers willing to participate enrolled by completing the consent forms and a baseline questionnaire and returned these in a pre-paid envelope to the study coordination center. Data at all sites were collected between January and December 2011.

In Nigeria, health workers were randomly selected from a sampling frame of health workers at two hospitals in Abuja, one urban and another peri-urban. Ugandan participants were enrolled from two sites, one in Wakiso District, a peri-urban community 30 min north of the capital Kampala, and the second in Bushenyi District, a rural south-western district. The study team first met with community and district leaders and provided information about the study widely to the community. Villages in the district were selected by a multi-stage sampling approach.

In western Uganda, the study team enumerated the number of households in the selected villages and then randomly selected households for inclusion. The interviewers conducted a door-to-door exercise to interview the head of household or consenting adult at the selected homes with the aim to achieve equal gender representation. Participants who agreed to participate signed an informed consent form and those unable to read or write had the form read to them followed by a thumb print to indicate willingness to participate.

In the peri-urban cohort of Wakiso District, two parishes consisting of 13 villages were randomly selected in Nangabo sub-county. Research assistants moved from house-to-house in these villages providing information about the study and explaining the objectives. They sought written consent from the head of household or the most responsible adult present to participate in the study. At both sites in Uganda, one consenting adult member was enrolled per household.

### Human subjects

Informed consent was obtained from each subject either by voluntarily posting back a signed form with a completed questionnaire (South Africa and Tanzania) or through documentation with trained interviewers (Nigeria and Uganda). The proposal was approved in the respective host country institutional review boards and at Harvard University.

### Measurements

Across the five sites, we developed and administered a core questionnaire that was standardized and lasted approximately 1 h. The questionnaire included sections on socio-economic status, infectious and chronic disease diagnoses, mental health and injuries, and behaviors including intimate partner violence (IPV), smoking, alcohol and use of other substances such as marijuana. English questionnaires were used in Nigeria and South Africa, and were translated and back translated into kiSwahili in Tanzania, Luganda and Runyakitara in Uganda, and into Afrikaans in South Africa.

We used a modified Beck Depression Inventory (BDI) scale with four items to measure DS as proposed by Hakstian & McLean (1989). The four items were: pre-occupied by thoughts of hopelessness, feeling relaxed, difficulty starting or following through a task and satisfaction with ability to perform regular duties. When tested in African populations, the BDI has been found to be valid and reliable (Ward *et al*. 2003; Adewuya *et al*. 2007). We asked about alcohol use and separately; we asked participants whether they had ever used any substances such as marijuana or methamphetamines to elevate their mood and these have collectively been defined as NAS. Therefore, the substances were classified as alcohol or NAS, and the NAS included marijuana and methamphetamines. Alcohol and NAS use were classified as dichotomous variables.

### Data analysis

Age was dichotomized at the 75th percentile to classify participants as young *v.* old. The primary outcome for this analysis was DS as a dichotomous outcome. The four items from the BDI scale were used to compute a final depression score. This composite score for depression was obtained using raw score discriminate function coefficients approximately proportional to 4, 1, 1 and 1, respectively, for the four items as described in the modified BDI scale (Hakstian & McLean, 1989). We used cut offs based on this paper, and according to the scale, the maximum score is 50. A score of 22 or more defines the presence of general psychopathology-related distress (PRD). A score of 25 or more is used to define possible depression or DS. A score of 21 or less was considered normal. We also explored both DS and PRD as study outcomes, in addition to NAS. Although it is an outcome variable, we explored NAS as an exposure variable to determine its association with DS based on previous studies (Saban *et al*. 2014). We excluded Tanzania in the analysis of PRD and DS because one of the items of the BDI scale was missing when the data collection was completed.

We used generalized estimating equation models (Williamson *et al*. 1996) using the *xtgee* command in STATA, with exchangeable correlation, to calculate the odds ratios (OR) for factors associated with the use of NAS. Because the frequency of PRD and DS was high, OR would overestimate the risk ratios for the factors associated with these outcomes. Also, our data were clustered by country of data collection. Therefore, we used random-effects logistic regression models to estimate the prevalence ratios (PR) for various exposure factors associated with the study outcomes (Li *et al*. 2011), with inclusion of a random intercept for each country. In the multiple regression, we aimed to build a parsimonious model, and aimed to use the minimum adjustable set of variables. We tested explanatory variables for correlation in order to avoid introduction of collinearity. For instance, gender and education were highly correlated (because of the dominance of women in nursing and teaching professions) and both could not be entered into the multiple regression model simultaneously. In the multiple regression analysis, the minimum adjustable set of variables included age, sex and alcohol use. All analyses were completed using STATA version 12 (College Station, Texas, USA).

## Results

We enrolled 1415 participants at the five sites. Of these, 489 (34.6%) were male. The mean age was 40.6 years for males and 41.6 years for females. Over 90% had access to a cell phone. The socio-demographic characteristics are shown in Table 2.

**Table 2.** Baseline characteristics of respondents in four sub-Saharan African countries by gender.

Characteristic	Total *n* = 1415	Male *n* = 489	Female *n* = 926	*p* Value for gender difference
Age (mean, s.d.)	41.2 (12.3)	40.6	41.6	0.22
Age categories				
<75th percentile (50.55 years)	755 (53.4)	289 (59.1)	466 (50.3)	
75th percentile and above	660 (46.6)	200 (40.9)	460 (49.7)	**0.002**
Site				
Uganda peri-urban	297 (21.0)	139 (28.4)	158 (17.1)	
Uganda rural	200 (14.1)	100 (20.4)	100 (10.8)	
Nigeria	200 (14.1)	67 (13.7)	133 (14.4)	
South Africa	489 (34.6)	145 (29.6)	344 (37.1)	**<0.001**
Tanzania	229 (16.2)	38 (7.9)	191 (20.6)	
Currently smoking	138 (10.1)	76 (15.7)	62 (6.9)	**<0.001**
Have cell phone access	1041(92.1)	400 (92.4)	641 (91.9)	0.80
Number of household members				
0	590 (51.9)	231 (52.8)	359 (51.3)	
1	484 (42.6)	176 (40.3)	308 (44.0)	
2	63 (5.5)	30 (6.9)	33 (4.7)	0.19
Education				
None	53 (3.9)	18 (3.8)	35 (4.1)	
Primary/secondary/vocational	631 (47.5)	230 (48.2)	401 (47.0)	
Post-secondary/college	645 (48.6)	228 (47.9)	417 (48.9)	0.88
Marital status				
Never married	243 (17.6)	89 (18.4)	154 (17.2)	
Married/cohabiting	973 (70.4)	362 (74.9)	611 (67.9)	
Separated/divorce/widow	166 (12.0)	32 (6.7)	134 (14.9)	**<0.001**
Number of children				
None	137 (12.1)	59 (14.9)	78 (10.5)	
One to two	397 (34.9)	119 (30.3)	278 (37.3)	
Three to four	353 (30.9)	106 (26.9)	247 (33.1)	
Five and above	252 (22.1)	110 (27.9)	142 (19.1)	**<0.001**
Alcohol use				
Never or <1 per month	731 (77.8)	271 (75.3)	460 (79.5)	
More than 1 per month	208 (22.2)	89 (24.7)	119 (20.6)	0.14
BMI category				
Underweight	24 (2.0)	11 (2.6)	13 (1.7)	
Normal	396 (32.8)	197 (46.7)	199 (25.3)	
Overweight	374 (30.9)	125 (29.6)	249 (31.7)	
Obese	414 (34.3)	89 (29.1)	325 (41.3)	**<0.001**
PRD^a^				
No	720 (60.7)	299 (66.3)	421 (57.3)	
Yes	466 (39.3)	152 (33.7)	314 (42.7)	**0.002**
Depressive symptoms^a^				
No	803 (67.7)	329 (72.9)	474 (64.5)	
Yes	383 (32.3)	122 (27.1)	261 (35.5)	**0.002**
Non-alcohol substance abuse				
No	1163(95.7)	416 (93.4)	747 (96.9)	
Yes	52 (4.3)	28 (6.3)	24 (3.1)	**0.008**

a Excludes Tanzania site.

p Values in bold are significant at 0.05 level.

### Study outcomes

Overall, 466 (39.3%) respondents had general PRD and the frequency of PRD was higher among women (42.7%) compared with men (33.7%), and this difference was statistically significant (*p*  =  0.002). DS occurred among 383 (32.3%) respondents, and like PRD, the occurrence was higher among women (35.5%) compared with men (27.1%) with χ^2^, *p*  =  0.002. Overall, use of NAS was reported among 52 (4.3%) respondents. The use was higher among men (*n*  =  28 or 6.3%) compared with the women (*n*  =  24 or 3.1%), and the difference was statistically significant (*p*  =  0.008).

### Factors associated with PRD

In the bivariate analysis, females were more likely to have PRD compared with men [PR 1.19, 95% confidence interval (CI) 1.16–1.91]. Other significant risk factors included alcohol use, IPV, larger number of children, poor rating of general health and having diabetes or heart disease as comorbidities. However, having a higher education was protective of PRD. Use of NAS was not associated with PRD (Table 3). In the multivariable analysis, only gender, alcohol use and IPV remained significant. The data analysis excluded Tanzania.

**Table 3.** Factors associated with PRD among respondents in four sub-Saharan African countries^a^.

Characteristic	Crude PR		Adjusted PR	
	PR (95% CI)	*p* Value	PR (95% CI)	*p* Value
Sex				
Male	1		1	
Female	**1.19 (1.16–1.91)**	**0.002**	**1.62 (1.19–2.19)**	**0.002**
Age				
<50.55 years	1		1	
50.55 years and above	**1.24 (0.92–1.66)**	**0.15**	1.33 (0.93–1.91)	0.12
Current smoker				
No	1		–	
Yes	1.10 (0.75–1.61)	0.62		
Cell phone access				
No	1		–	
Yes	0.91 (0.58–1.44)	0.69		
Use drugs to elevate mood				
No	1		–	
Yes	1.57 (0.88–2.82)	0.13		
Marital status				
Single	1			
Married	1.17 (0.84–1.62)	0.35	–	
Separated/divorced/widow	1.56 (0.99–2.44)	0.052		
Education				
None	1			
Primary/secondary/vocational	**0.41 (0.22–0.73)**	**0.003**	–	
Post-secondary/college	**0.28 (0.14–0.58)**	**0.001**		
Household members				
0	1			
1	**1.42 (1.09–1.84)**	**0.008**	–	
2	1.26 (0.73–2.18)	0.41		
Number of children				
None	1			
1–2	1.45 (0.92–2.29)	0.11		
3–4	1.47 (0.92–2.35)	0.11		
5 and above	**2.16 (1.34–3.48)**	**0.002**		
Alcohol use				
Never or <1 per month	1		**1**	
More than 1 per month	**1.43 (0.99–2.07)**	**0.056**	**1.52 (1.04–2.23)**	**0.03**
Intimate partner violence				
No	1		1	
Yes	**3.09 (1.75–5.47)**	**<0.0001**	**3.73 (1.87–7.44)**	**<0.001**
General health rating				
Good to very good	1		–	
Moderate to very bad	**2.98 (1.56–5.71)**	**0.001**		
Difficulty with work/household				
None-mild	1		–	
Moderate to extreme	**3.11 (2.26–4.26)**	**<0.001**		
Diagnosis of diabetes				
No	1			
Yes	1.96 (1.13–3.37)	**0.015**		
Diagnosis of heart disease				
No	1		–	
Yes	**2.29 (1.20–4.36)**	**0.012**		
BMI category				
Underweight				
Normal	0.82 (0.34–1.96)	0.65	–	
Overweight	0.82 (0.33–1.99)	0.66		
Obese	1.25 (0.51–3.07)	0.63		

*p* Values in bold significant at 0.05 level.

a Data analysis excludes Tanzania.

### Factors associated with DS

In the bivariate analysis, females were more likely to suffer from DS compared with males but age and alcohol use were not associated with DS. Use of NAS was significantly associated with DS (PR 1.97, 95% CI 1.08–3.59). IPV, poor rating of health and presence of comorbidities of diabetes or heart disease were all associated with increased odds of DS (Table 4). In the multivariable analysis gender, use of NAS and IPV remained significantly associated with DS. The analysis excluded Tanzania.

**Table 4.** Factors associated with depressive symptoms among respondents in four countries in sub Saharan Africa^a^.

Characteristic	Crude PR	Adjusted PR		
	PR (95% CI)	*p* Value	PR (95% CI)	*p* Value
Sex				
Male	1		1	
Female	**1.49 (1.14–1.94)**	**0.003**	**1.49 (1.11–2.02)**	**0.008**
Age				
<50.55	1		1	
50.55 and above	1.21 (0.88–1.64)	0.24	1.27 (0.91–1.78)	0.15
Current smoker				
No	1		–	–
Yes	1.08 (0.72–1.61)	0.72		
Cell phone access				
No	1			
Yes	0.96 (0.59–1.56)	0.87	–	–
Use drugs to elevate mood				
No	1			
Yes	**1.97 (1.08–3.58)**	**0.026**	**2.06 (1.11–3.85)**	**0.02**
Marital status				
Single	1			
Married	1.07 (0.77–1.52)	0.67	–	–
Separated/divorced/widow	1.13 (0.69–1.82)	0.62		
Education				
None	1			
Primary/secondary/vocational	**0.32 (0.18–0.58)**	**<0.001**	–	–
Post-secondary/college	**0.23 (0.11–0.47)**	**<0.001**		
Household members				
0	1			
1	**1.39 (1.06–1.83)**	**0.02**	–	–
2	1.39 (0.79–2.47)	0.25		
Number of children				
None	1			
1–2	1.38 (0.84–2.28)	0.19		
3–4	1.41 (0.85–2.34)	0.18	–	–
5 and above	**1.91 (1.11–3.28)**	**0.02**		
Alcohol use				
Never or <1 drink per month	1			
More than 1 drink per month	**1.33 (0.91–1.96)**	**0.14**		
Intimate partner violence				
No	1			
Yes	**2.99 (1.72–5.21)**	**<0.001**	**2.93 (1.61–5.37)**	**<0.001**
General health rating				
Good to very good	1			
Moderate to very bad	**2.01 (1.01–3.97)**	**0.04**		
Difficulty with work/household				
None to mild	1			
Moderate to extreme	**2.85 (2.05–3.95)**	**<0.001**	–	–
Diagnosis of diabetes				
No	1			
Yes	**2.03 (1.16–3.54)**	**0.013**	–	–
Diagnosis of heart disease				
No	1			
Yes	**2.18 (1.13–4.21)**	**0.019**	–	–
BMI category				
Underweight	1			
Normal	0.77 (0.31–1.98)	0.59		
Overweight	0.81 (0.32–2.09)	0.66	–	–
Obese	1.28 (0.49–3.34)	0.61		

*p* Values in bold are significant at 0.05 level.

a Data excludes Tanzania.

### Factors associated with NAS use

Females and older respondents were both less likely to report use of NAS. Current smokers and those reporting IPV were also more likely to use NAS. The results are shown in Table 5. In the multivariable analysis, female gender, older age and education were significantly protective for NAS use. However, current smokers and respondents who experienced IPV were more likely to use NAS compared with non-smokers and those who had not experienced IPV, respectively.

**Table 5.** Factors associated with use of drugs to elevate mood (NAS) among respondents in four sub-Saharan African countries.

Characteristic	Crude odds ratios	Adjusted odds ratios^a^		
Sex	OR (95% CI)	*p* Value	OR (95% CI)	*p* Value^b^
Male	1.00		1	
Female	**0.41 (0.19–0.85)**	**0.017**	**0.48 (0.25–0.95)**	**0.036**
Age (years)				
<50.55	1.00		1	
50.55 and above	**0.34 (0.17–0.66)**	**0.002**	**0.31 (0.18–0.52)**	**<0.001**
Current smoker				
No	1.00		1	
Yes	**3.47 (2.24–5.39)**	**<0.001**	**2.98 (2.12–4.21)**	**<0.001**
Cell phone access				
No	1.00			
Yes	0.76 (0.21–2.82)	0.69	–	
Marital status				
Single	1.00			
Married	0.98 (0.61–1.56)	0.92	–	
Separated/divorced/widow	1.75 (1.26–2.45)	0.001		
Education				
None	1.00		1	
Primary/secondary/vocational	0.31 (0.05–1.83)	0.19	0.33 (0.055–1.97)	0.23
Post-secondary/college	0.84 (0.075–9.55)	0.89	0.87 (0.088–8.67)	0.23
Alcohol use				
Never or <1 drink per month	1.00		–	
More than 1 drink per month	1.68 (0.97–2.91)	0.06		
Intimate partner violence				
No	**1.00**		**1**	
Yes	**2.33 (1.11–4.95)**	**0.026**	**2.16 (1.11–4.25)**	**0.024**
General health rating				
Good to very good	1.00		–	
Moderate to very bad	1.11 (0.98–1.27)	0.087		

*p* Values in bold are significant at 0.05 level

a Odds ratios calculated in a multiple logistic regression model with sex, age, current smoking status, site and educational level in the model.

b All other factors seen in Table 2 above were also explored but were not significant.

## Discussion

Our study, conducted in four countries in sub-Saharan Africa, shows that the prevalence of possible depression or DS is higher than in other surveys among non-HIV-infected populations on the continent. A recent study among female heads of households in Mozambique showed that depression was 14% (Audet *et al*. 2017), much lower than the 35.5% among the women in our study. However, it may be difficult to compare the occurrence of depression across different studies because different tools have been used to measure depression. In our study, we used a brief BDI scale, whereas the Mozambican study used the PHQ-8 tool (Kroenke *et al*. 2009).

Our study also showed a significant association between IPV and DS. This finding is in agreement with several other studies in sub-Saharan Africa (Tanimu *et al*. 2016; Kapiga *et al*. 2017; Peltzer & Pengpid, 2017). Literature suggests that IPV may lead to depression and some interventions have been proposed to prevent IPV (Kapiga *et al*. 2017) in order to curb depression, but generally there is limited effort on the continent to tackle this public health problem (Chepuka *et al*. 2014).

Our data showed that females were more likely to suffer DS. These findings are in agreement with what is generally known and confirm that women are at a higher risk for possible depression (Cole & Dendukuri, 2003; Anstey *et al*. 2007; Desouky & Allam, 2017). Contrary to the findings in these studies, our data showed that older age was not significantly associated with DS. We did not ask our respondents whether they had sought treatment for mental illness. Studies in sub-Saharan Africa show that there is a huge burden of undiagnosed and untreated mental illness due to a limited availability of these services (Azale *et al*. 2016; Kako *et al*. 2016).

NAS use was reported in <5% of the respondents. Although this seems low, substance use is on the rise and projected to rise along with mental illness to more than double the current prevalence by 2050 (Charlson *et al*. 2014). Use of NAS was more likely to occur among the younger population, who are expected to be the most productive in society. Our population was comparatively older, with a median age of 42 years. Hence, this may explain the low use in this older population. Our study showed a significant association between NAS use and DS. Our findings are in agreement with several other studies to show that NAS use is associated with mental illness (Saban & Flisher, 2010; Saban, *et al*. 2014; Dierker *et al*. 2018).

Awareness of the tsunami of non-communicable disease now hitting the sub-Saharan population is slowly growing. However, large-scale efforts to reveal the specific causes – genetic and environmental – in this population are lacking, which hampers evidence-based prevention efforts. Our pilot study has undoubtedly a suboptimal cross-sectional design, is limited in size and investigates only five selected populations in four countries. Nevertheless, it has convincingly documented that the burden of NCDs is already enormous (Dalal *et al*. 2011; Guwatudde *et al*. 2015; Ajayi *et al*. 2016; Chiwanga *et al*. 2016) and that the need for action is urgent (Holmes *et al*. 2010). A conservative extrapolation of results presented in this study does suggest that at least 160 million individuals in the sub-Saharan population suffer from depression with minimal or no access to adequate medical treatment.

Our study participants were drawn from diverse groups of populations in sub-Saharan Africa ranging from rural subsistence farmers in Uganda, to teachers in South Africa and health workers in Nigeria. The strength in the diversity is that the results can be widely generalizable given the differences in lifestyle and risk exposure. However, it is important to note that small sample sizes were drawn from each of the subgroups. For this reason, our results do not explicitly discuss and compare findings between the difference countries. To reasonably capture between country differences, much larger studies should be conducted.

One of the strengths of our study is that it is not based in health facilities, nor was it limited to HIV-infected populations. Secondly, our study population is drawn from a diverse group of populations in sub-Saharan African, ranging from rural population in Uganda to professionals in South Africa. Our study has some weaknesses: this is a cross-sectional design, hence we are not able to establish temporal relationships and causal links. It is indeed difficult to establish whether the DS came before the IPV or *vice versa*. In addition, we used brief questions to measure partner violence and substance abuse rather than complete validated tools. We recommend that longitudinal studies should be conducted to establish stronger evidence, rigorously measure the risk factors with standard tools and therein design interventions (Holmes *et al*. 2010). Our study provides preliminary evidence to inform these cohort studies and randomized controlled intervention studies.

## Conclusion

In conclusion, our study in four countries in sub-Saharan Africa has shown that the prevalence of DS is very high. It is related to NAS use and IPV. Screening for depression among those reporting IPV and NAS use among the younger persons will identify those who most need intervention.
